# Adduction to arginine detoxifies aflatoxin B1 by eliminating genotoxicity and altering *in vitro* toxicokinetic profiles

**DOI:** 10.18632/oncotarget.23382

**Published:** 2017-12-17

**Authors:** Blake R. Rushing, Mustafa I. Selim

**Affiliations:** ^1^ Department of Pharmacology and Toxicology, East Carolina University, Brody School of Medicine, Greenville, NC, 27834, USA

**Keywords:** aflatoxin, adduction, genotoxicity, ADME, mass spectrometry

## Abstract

Aflatoxin B1 (AFB1), a class 1 carcinogen and prominent food contaminant, is highly linked to the development of hepatocellular carcinoma (HCC) and plays a causative role in a large portion of global HCC cases. We have demonstrated that a mixture of common organic acids (citric and phosphoric acid) along with arginine can eliminate >99% of AFB1 in solution as well as on corn kernels and convert it to the AFB2a-Arg adduct, acting as a potential detoxification process for contaminated foods. Evaluation of toxicokinetic changes after AFB2a-Arg formation show that the product is highly stable in biological fluids, is not metabolized by P450 enzymes, is highly plasma protein bound, has low lipid solubility, and has poor intestinal permeability/high intestinal efflux compared to AFB1. Ames’ test results show that at mutagenic concentrations of AFB1, AFB2a-Arg does not have any measurable mutagenic effect which was confirmed by DNA adduct identification by liquid chromatography-mass spectrometry. Evaluation in HepG2 and HepaRG cells showed that AFB2a-Arg did not cause any significant decreases in cell viability nor did it increase micronuclei formation when administered at toxic concentrations of AFB1. These results show that conversion of AFB1 to AFB2a-Arg is a potential strategy to detoxify contaminated foods.

## INTRODUCTION

Aflatoxins are a group of mycotoxins produced by the fungi *Aspergillus flavus* and *Aspergillus parasictus* which frequently contaminate many varieties of the world's crops, particularly maize, groundnuts, and wheats [1–5]. Out of the compounds belonging to this class, aflatoxin B1 (AFB1) is the most toxic of the aflatoxins. As a class 1 carcinogen, chronic dietary exposure to AFB1 has been identified as a major risk factor for the development of hepatocellular carcinoma (HCC) particularly when an individual is also infected with hepatitis B virus [6–10]. AFB1 initiates the development of HCC through mutagenesis which occurs after it is metabolized in the liver to aflatoxin-8,9-epoixde (AFBO), a bioactive metabolite which spontaneously binds irreversibly to guanine residues forming AFB1-N_7_-Guanine adducts which lead to the development of point mutations [11, 12]. In addition to HCC, links between AFB1 exposure and growth suppression as well as immune system modulation have been shown, amplifying its status as a public health concern [13–15]. Sub-Saharan Africa and Southeast Asia are the regions which possess the highest risk of exposure to aflatoxins and have experienced aflatoxin outbreaks severe enough to cause hundreds of deaths due to acute toxicities [16–18]. While these areas experience the greatest burden of aflatoxins, it is predicted that as the average global temperature increases due to climate change, many countries may experience greater risks of aflatoxin exposure in the future [19].

Many strategies to manage aflatoxin contamination in crops have been studied to alleviate global exposure rates. Because contamination can occur at both the pre- and post-harvest stage, these approaches have included various agricultural practices (i.e. irrigation/pesticide practices, use of genetically modified seeds which are mold-resistant), drying and storage techniques, as well as various food processing strategies [20]. Due to the unpredictable timing of contamination, food processing techniques have gained popularity due to the ability to reduce exposure just prior to ingestion. These often incorporate chemoprotection, enterosorption, acidification/alkalization, or other methods of chemical treatment to inactivate, modify, or remove AFB1 [21, 22]. Many of these methods have limited efficacy or other logistical limitations that have prevented their use by various populations (i.e. necessitates high cost equipment, requires chronic dosing of individuals with medications/supplements). Additionally, transformation products that may arise from these detoxification methods may still retain genotoxic activity or even be reversible back to AFB1 [23, 24], demonstrating that transformation products must be fully evaluated to ensure detoxification. As a result, there is a large need for a decontamination method that is effective, forms inert products, and is relatively easy to implement into societies of varying economic status.

**Figure 1:**
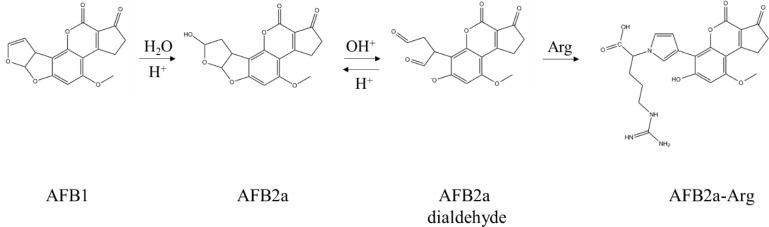
Chemical conversion of AFB1 to AFB2a-Arg is carried out by the AFB2a intermediate Formed by hydration of AFB1 under acidic conditions, AFB2a spontaneously converts to a dialdehyde which is favored under alkaline conditions. This AFB2a dialdehyde is then able to react with the primary amine group of arginine through the formation of a pyrrole ring.

Recently, our lab has investigated the efficacy of common organic acids or acidic environmental conditions to transform AFB1 into aflatoxin B2a (AFB2a) - an AFB1 metabolite which has shown to have reduced mutagenicity due to hydration of the 8,9-double bond [25–27]. Although *in vivo* data is limited, AFB2a has still been shown to have some hepatotoxic effects at high doses and there is a concern for its ability to spontaneously dehydrate back to AFB1, so its role as a detoxification end-product is unclear [28]. One property of AFB2a that likely contributes to its hepatotoxic effects is protein binding which, in a second study, we have shown that this adduct forms through the creation of a pyrrole ring [29]. Formerly thought to be formed through Schiff bases, this AFB2a-amine adduct is more stable than previously suggested and can be formed under mild acidic conditions. In this study, we hypothesized that AFB1 could be transformed into AFB2a and then subsequently adducted to an amino acid in a single acidification step. We also hypothesized that this reaction product would not be genotoxic due to the irreversible removal of the 8,9-double bond and/or by changing various toxicokinetic parameters to prevent the product from reaching the liver. Based on our previous work, arginine was chosen as the amino acid for adduction based on its high polarity previously determined by HPLC and the high pKa of its retained side chain which would remain charged at physiological pHs (Figure 1). These properties were expected to decrease the adduct's affinity for nonpolar environments, decreasing its lipid solubility and membrane permeability thereby increasing its effectiveness as a detoxification product.

## RESULTS

### Development of a treatment method to detoxify AFB1

**Figure 2:**
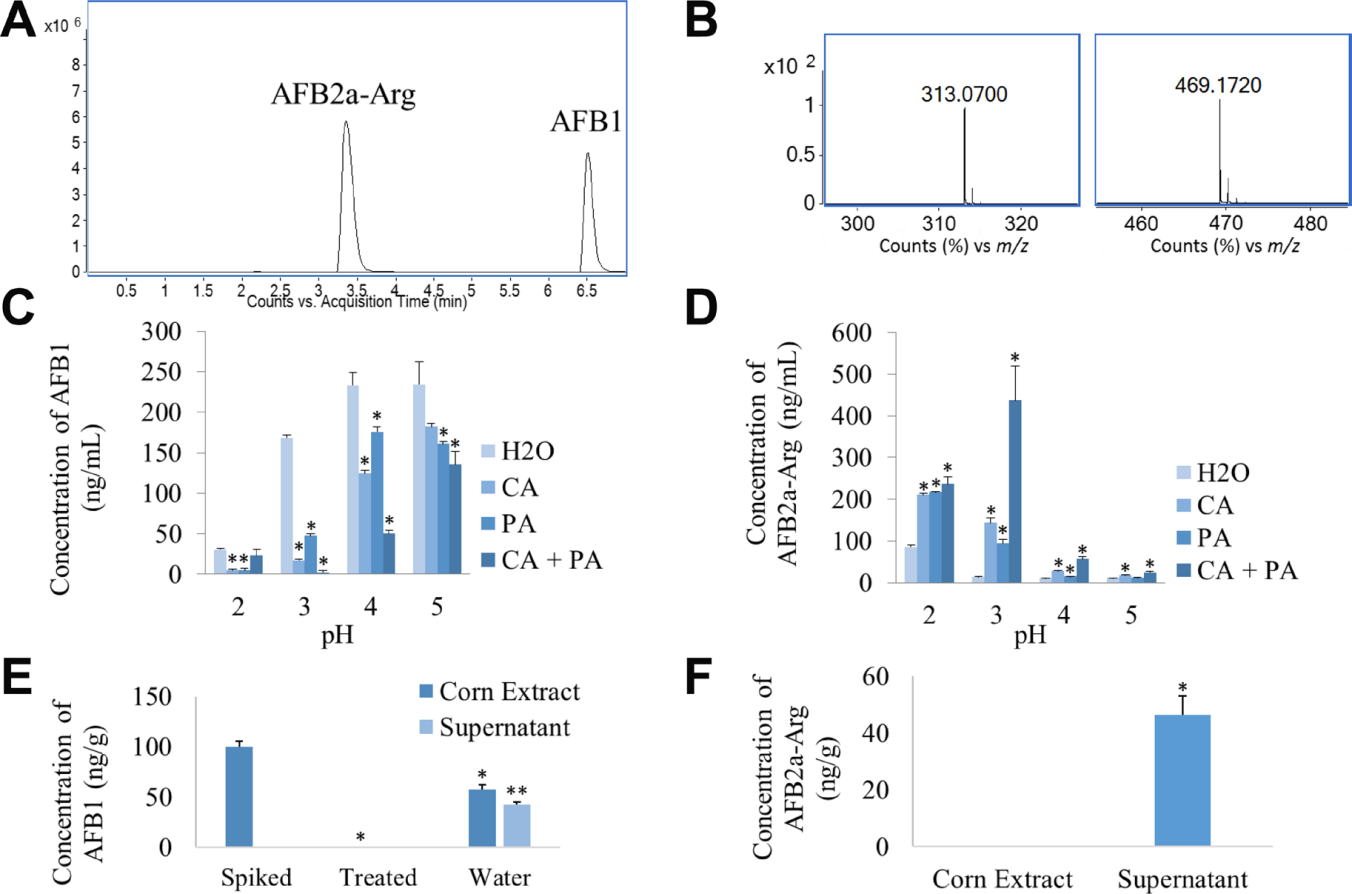
AFB1 can be converted to AFB2a-Arg in a single step using solutions containing organic acids and arginine AFB2a-Arg standards were distinguished from AFB1 standards and quantified using extracted ion chromatograms (**A**) which were formed based on accurate mass spectra (**B**) (left – AFB1, right – AFB2a-Arg) to develop an HPLC-MS method. Standards of AFB1 were dissolved in arginine solutions containing various combinations of citric and/or phosphoric acid at various pH levels. After boiling for 20 min, disappearance of AFB1 (**C**) and formation of AFB2a-Arg (**D**) was observed. Taking the optimal conditions from this experiment, corn kernels spiked with AFB1 were treated and then analyzed for AFB1 (**E**) and AFB2a-Arg (**F**) that either remained on the corn or was dissolved in the treatment solution. All error bars are expressed as SEM. Asterisks denote statistical significance from the control (*p <* 0.05).

Because AFB1 can be transformed into AFB2a which can then adduct to amines under acidic conditions, we investigated the transformation of AFB1 into AFB2a-Arg in a single treatment step across multiple acidic pH values. In previous work, we found that by adding various concentrations of citric acid into simulated gastric fluid, the transformation of AFB1 into AFB2a was greatly enhanced even though the pH remained unchanged [25]. Therefore, we also tested the effect of having either citric acid, phosphoric acid, or both present in the solution at each pH level. Using HPLC-MS, we were able to develop a method to detect the presence of AFB1 as well as AFB2a-Arg as the detoxification product after boiling each treatment solution for 20 min (Figure 2A and 2B). In general, as pH decreased, the amount of AFB1 remaining in solution decreased. The presence of citric and phosphoric acid, particularly in combination, enhanced AFB1 degradation even though the pH of the solution was the same as the H_2_O controls (Figure 2C). In accordance with AFB1 degradation, AFB2a-Arg production was greatest in solutions at lower pH values and with those that contained the organic acids, especially in combination. The most effective treatment solution, which degraded the most AFB1 and formed the most AFB2a-Arg, was one that contained both acids and was adjusted to a pH value of 3 (Figure 2D).

Because this composition was most effective on AFB1 standards, it was also tested for its ability to detoxify AFB1-spiked corn kernels. After treatment was applied to the contaminated corn, no detectable AFB1 was found on the corn surface or in the treatment solution itself. Some removal of AFB1 from the food surface could be attributable to water solubility of AFB1 as shown by the water washed group. However, this only removed approximately 40% of AFB1 which was found in the water solution itself untransformed (Figure 2E). When analyzing the treated corn, the detoxification product AFB2a-Arg was only found in the treatment solution and none was detected on the food substrate (Figure 2F). This shows that the treatment solution was not only able to remove AFB1 from the contaminated food, but it was also able to fully transform it into the arginine adduct.

### Toxicokinetic differences between AFB1 and AFB2a-Arg

**Figure 3:**
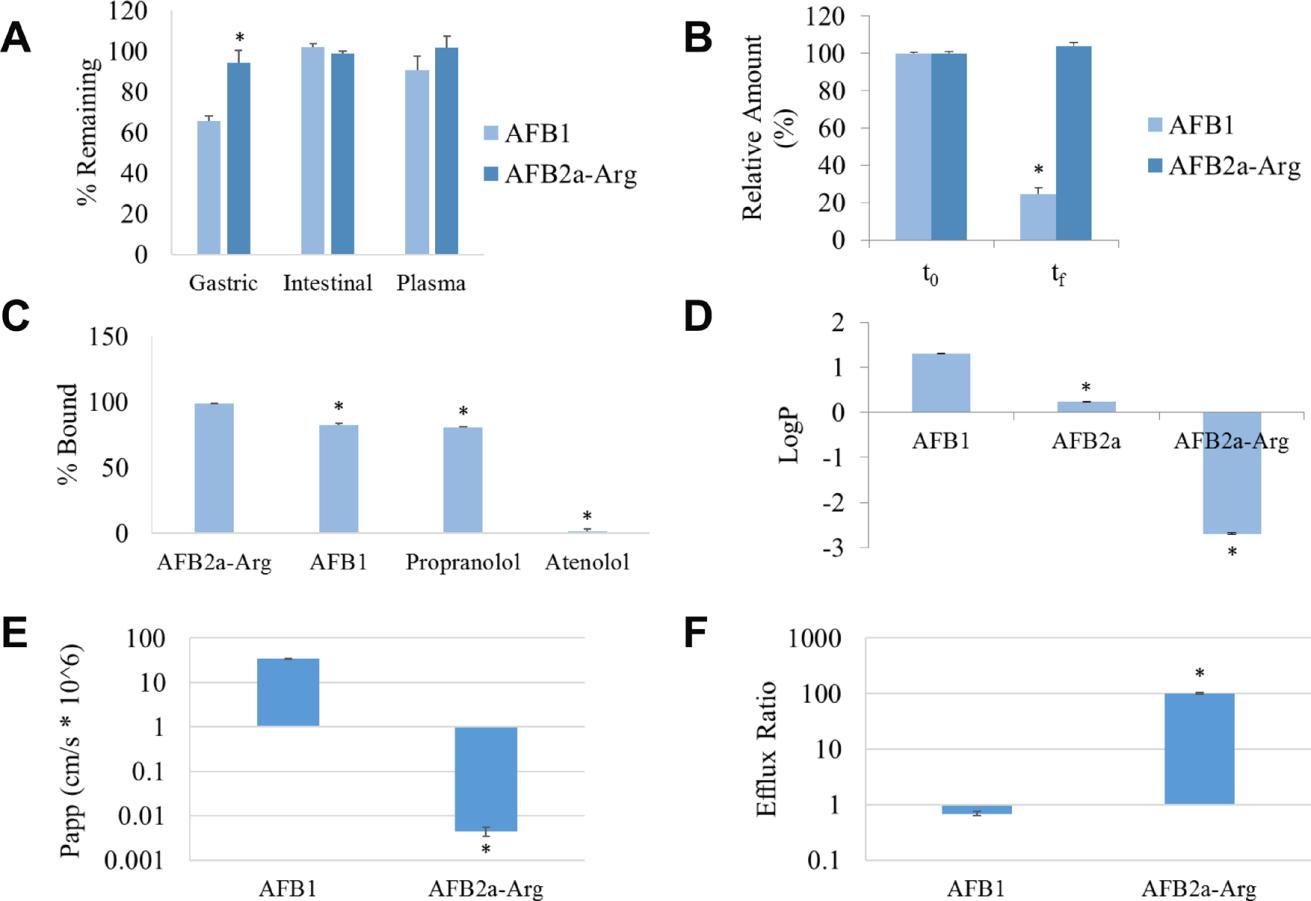
Conversion of AFB1 to AFB2a-Arg leads to changes in toxicokinetics The stability of AFB1 and AFB2a-Arg in biological fluids (**A**) and with hepatic microsomes (**B**) showed that AFB2a-Arg was more stable in acidic environments and was not metabolized by P450 enzymes. RED experiments showed that AFB2a-Arg was more highly protein-bound (**C**) and logP measurements demonstrated much poorer lipid solubility than AFB1 (**D**). Lastly, Caco-2 permeability assays showed that AFB2a-Arg had significantly less intestinal permeability (**E**) and higher intestinal efflux capacity (**F**) than AFB1. All error bars are expressed as SEM. Asterisks denote statistical significance from the control (*p <* 0.05).

A series of *in vitro* ADME tests were applied to AFB1 and AFB2a-Arg to determine any favorable toxicokinetic changes that may result from the transformation process. When incubated in simulated gastric fluid, simulated intestinal fluid, or pooled human plasma, AFB2a-Arg remained approximately 94%, 99%, and 102% stable in all compartments. Furthermore, AFB2a-Arg was significantly more stable than AFB1 in simulated gastric fluid which was hydrated into AFB2a under the low pH conditions (Figure 3A). These data indicate that AFB2a-Arg is stable in all three biological compartments, evading breakdown by hydrolysis or hydration. AFB2a-Arg was not significantly degraded by hepatic microsomal enzymes in contrast to AFB1 which was degraded by approximately 75% (Figure 3B). This lack of microsomal degradation shows that AFB2a-Arg is not a substrate for phase I metabolism, preventing hepatic oxidation into epoxides or other reactive metabolites.

Plasma protein binding measured by rapid equilibrium dialysis (RED) showed that AFB2a-Arg was 99% protein bound as opposed to AFB1 which was only 82% bound (Figure 3C). The highly polar, positively charged arginine side chain of AFB2a-Arg likely interacts very strongly with acidic residues of plasma proteins, increasing the strength of the interaction between the two. High affinity to plasma proteins decreases the portion of free AFB2a-Arg and could limit any biological effects it may have. However, the elimination rates may also be decreased due to this property, possibly increasing the biological half-life as compared to AFB1. When evaluating octanol-water partition coefficients, AFB2a-Arg showed very little partitioning into the octanol fraction leading to a lower partition coefficient than AFB1 or AFB2a (Figure 3D). This lowered lipid solubility suggests that AFB2a-Arg would have a greater difficulty passing through membranes particularly through passive diffusion. In agreement with this, the intestinal permeability of AFB2a-Arg, determined by the Caco-2 permeability assay, was also shown to be significantly lower than AFB1 (Figure 3E). Such poor intestinal permeability indicates that upon ingestion, AFB2a-Arg is much less likely to be absorbed into systemic circulation than AFB1. This is further highlighted by the observation that AFB2a-Arg demonstrated a significantly higher efflux ratio than AFB1 in Caco-2 cells (Figure 3F) which indicates that if any AFB2a-Arg is absorbed, a large portion of it will likely be effluxed back out into the lumen or extracellular space.

### Loss of mutagenicity after transformation of AFB1 into AFB2a-Arg

**Figure 4:**
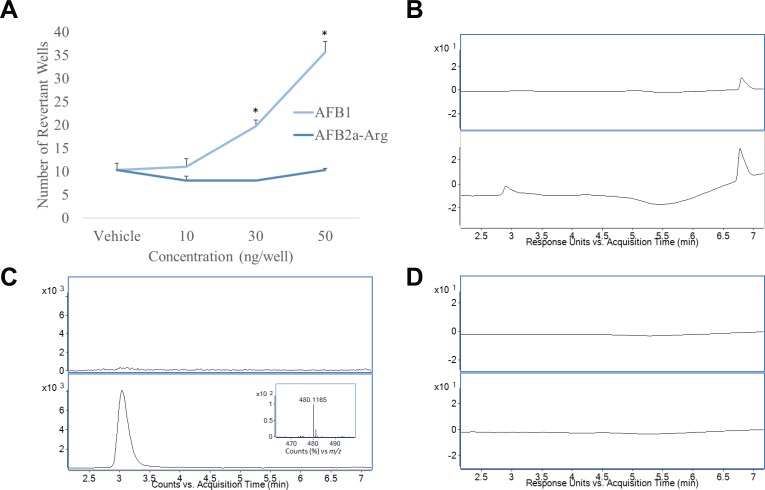
Conversion of AFB1 to AFB2a-Arg removes mutagenicity through eliminating DNA adduct formation Ames’ test results showed that at equivalent concentrations, AFB2a-Arg did not cause mutations although AFB1 showed a robust response (**A**). Incubations of AFB1 with DNA and liver microsomes showed the formation of a unique peak (2.9 min) in the HPLC UV chromatogram (**B**) which corresponded to the extracted ion chromatogram of the accurate mass of AFB1-N_7_-Guanine (**C**) (accurate mass spectra of AFB1-N_7_-Guanine shown in insert). Incubation of AFB2a-Arg with DNA and liver microsomes showed no unique peaks in the HPLC UV chromatogram indicating no DNA adduct formation (**D**). All error bars are expressed as SEM. Asterisks denote statistical significance from the control (*p <* 0.05).

To compare the mutagenic potential of AFB1 and the newly formed AFB2a-Arg, both compounds were evaluated by an Ames’ test and by their ability to form adducts with DNA as determined by HPLC-MS. In agreement with previous studies [27], Ames’ test results showed that at concentrations of 30 and 50 ng/well, AFB1 showed a significant increase in the number of revertant wells indicating a robust mutagenic response. In contrast, AFB2a-Arg did not show any significant differences from the vehicle at the same concentrations (Figure 4A). When incubated with DNA and liver microsomes, UV chromatograms showed the formation of a new peak that absorbed strongly at 365 nm (Figure 4B). Upon analyzing mass spectral data for this peak, the accurate mass of AFB1-N_7_-Guanine (*m/z* 480.115) was discovered which gave a strong peak in the extracted ion chromatogram indicating the presence of DNA adduction (Figure 4C). This *m/*z has also been previously used to identify AFB1-N_7_-Guanine and has been correlated to AFB1 exposure and toxicity [33, 34]. When AFB2a-Arg was incubated with DNA and liver microsomes, no additional peaks were discovered in the UV chromatograms (Figure 4D) and mass spectral data showed no accurate masses that would correspond to any theoretical DNA adducts (data not shown). These data indicate that AFB2a-Arg does not show any mutagenic potential at concentrations that show clear, robust responses with AFB1.

### Genotoxicity and cytotoxicity of AFB1 and AFB2a-Arg in human hepatocyte cell lines

**Figure 5:**
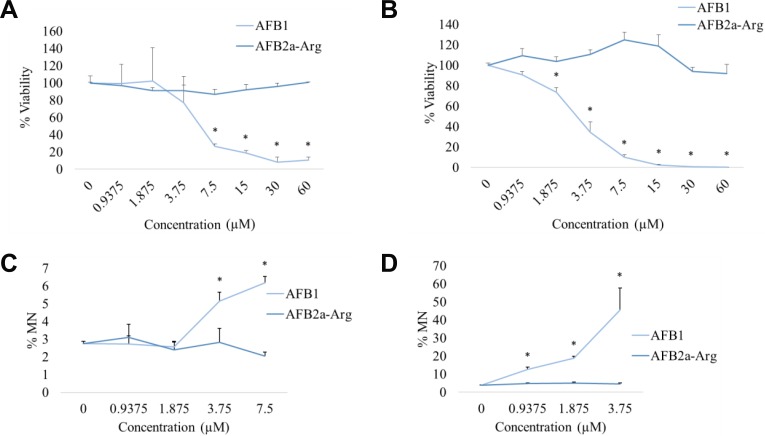
Cytotoxicity and genotoxicity measurements in cultured human hepatocyte cell lines Cell viability was measured in HepG2 (**A**) and HepaRG (**B**) by flow cytometry after 24 hours of treatment with AFB1 or AFB2a-Arg followed by 72 hours of recovery. Using the same treatment paradigm, the percentage of cells containing micronuclei (MN) was also measured by flow cytometry in HepG2 (**C**) and HepaRG (**D**) cells as a measure of genotoxicity. Both endpoints showed that AFB2a-Arg, in contrast to AFB1, caused no hepatocyte cell death or micronuclei formation. All error bars are expressed as SEM. Asterisks denote statistical significance from the control (*p <* 0.05).

To determine hepatotoxic effects of AFB2a-Arg with AFB1, genotoxicity and cytotoxicity were measured after exposing HepG2 and HepaRG cells to both compounds. Results showed that by observing cell viability, AFB1 significantly reduced the percentage of viable cells at concentrations >7.5 μM and >3.75 μM for HepG2 and HepaRG cells respectively. In contrast, AFB2a-Arg showed no significant decreases in cell viability in all concentrations tested (Figure 5A and 5B). When analyzing concentrations that contained >25% cell viability for both AFB1 and AFB_2a_-Arg treated cells, AFB1 treated cells showed a significant increase in the number of micronuclei in both HepG2 and HepaRG cell lines whereas AFB2a-Arg did not show this genotoxic marker (Figure 5C and 5D). Although micronuclei percentage at higher concentrations are not displayed due to high AFB1 induced cell death, AFB_2a_-Arg also did not increase micronuclei formation all the way up to the 60 μM used in this study. The effects of AFB1 at the dosages used in this study are comparable to genotoxic responses shown in previous studies using both HepG2 and HepaRG cell lines [35–37]. The higher metabolic activity of HepaRG cells likely contributes to the increased sensitivity and range of micronuclei that were observed as compared to HepG2 cells. Taken together, these data show that AFB2a-Arg does not lead to genotoxicity in human liver cell lines. Additionally, the absence of cytotoxicity also supports a lack of overall hepatotoxicity of AFB2a-Arg.

## DISCUSSION

Various studies have shown that food supplies in many countries are contaminated with high levels of AFB1. Ingestion of AFB1 through these contaminated foods has been strongly linked with carcinogenicity and it does so through damaging genetic material [38]. As a result, treatment of contaminated foods has been a widely studied area and many strategies involve methods that chemically modify AFB1. Some studies simply look at AFB1 disappearance, however this is not sufficient evidence of detoxification because some transformation products may still retain the original compound's toxicity. Additionally, many of these transformation products are also not evaluated for kinetic properties such as metabolism or bioavailability. Also, the treatment methods themselves have various limitations such as insufficient efficacy or logistical issues regarding practical use [39]. As a result, there is still a great need to discover new methods to detoxify contaminated foods.

**Figure 6:**
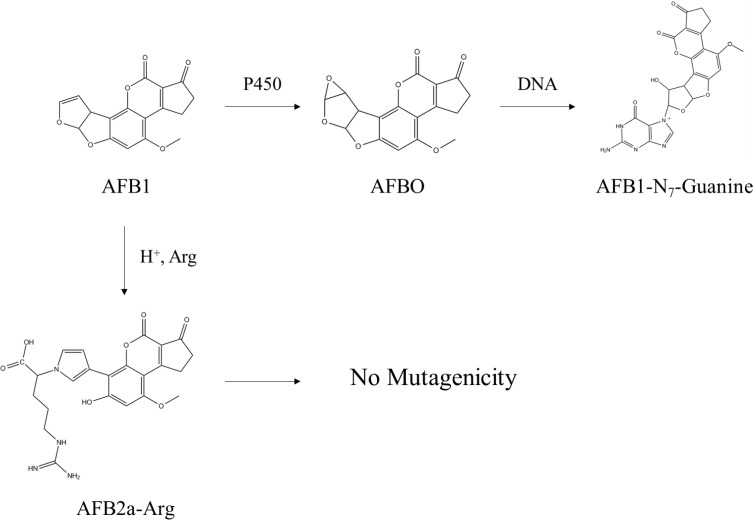
Summary of AFB1 detoxification through arginine adduction By using acidic solutions containing arginine, AFB1 can be converted prior to ingestion to AFB2a-Arg which had no measurable genotoxic effects and had favorable changes in toxicokinetic properties which would reduce liver tissue concentrations. This conversion prevents AFB1 from progressing down its natural genotoxic pathway of bioactivation to AFBO and subsequent binding to DNA at guanine residues.

In this study, we explored the possibility to transforming AFB1 into an amino acid adduct and the changes this transformation has on the toxicokinetic or toxicodynamic properties of the original AFB1. Our findings show that after treating contaminated foods with a mixture of organic acids and arginine at acidic pH values, a full conversion of AFB1 into AFB2a-Arg can be achieved in 20 minutes without the use of any specialized equipment. A panel of *in vitro* ADME tests were applied to both compounds and the data showed that AFB2a-Arg was highly stable, was not metabolized, had a reduced ability to be absorbed through the intestine, and had a lowered ability to distribute to target organs due to high plasma protein binding. Lastly, AFB2a-Arg showed no mutagenic, genotoxic, or cytotoxic potential in bacterial cells or in cultured human hepatocytes. Taken together, the data suggest that AFB2a-Arg can be easily formed from AFB1 and the transformation is an effective method to reduce AFB1 toxicity (Figure 6).

AFB2a-Arg formation was enhanced in the presence of organic acids, at constant pH values, even though protonation is the rate limiting step in hydration reactions of alkenes. One would expect that the solution pH itself should be the determining factor in the conversion of AFB1 to AFB2a regardless of other dissolved species. However, the observation seen in this study is in agreement with our previous findings concerning AFB2a formation in gastric fluid containing citric acid [25]. It could be that the presence of the organic acids helps to buffer the treatment solution during boiling to keep the pH constant, or perhaps the conjugate bases of these acids stabilize the intermediate species to enhance the hydration. Exploring the use of other buffering species in this treatment process may be useful in further optimization of this detoxification. Additionally, longer boiling times may be used with lower acid concentrations to achieve the same effect. These options may lead to even cheaper or convenient methods to treat contaminated foods.

The approach for the current study used *in vitro* methods to assess our detoxification product, however *in vivo* methods may useful in confirming or expanding this evaluation. Such information would useful to further explore the bioavailability of AFB2a-Arg as well as its excretion. Additionally, AFB2a-Arg may be a substrate for other enzymes outside of the liver microsomal fraction used in this study. Furthermore, other toxic endpoints may be evaluated using these models to determine if AFB2a-Arg has other non-genotoxic effects.

Another important aspect of treatment methods is their effect on the nutritional value of the foods to which they are applied. Oftentimes, these methods can vary the quantity of macronutrients or limit their absorption into the body [40–42]. Because AFB1 contamination commonly occurs in regions with high rates of malnutrition, it is important to maintain the integrity of the nutritional value of the detoxified food. Although this aspect of the treatment method was not evaluated in this study, organic acids have been extensively used on foods, usually as preservatives, nutritional supplements, or as naturally occurring constituents and have not been associated with reducing nutritional quality [43–51]. Regardless, a complete evaluation of this treatment process on the integrity of various foods commodities will be important towards establishing its practical use worldwide.

## METHODS

### Materials

Standards of aflatoxins, propranolol HCl, atenolol, LCMS grade solvents, L-arginine, citric acid, phosphoric acid, calf thymus DNA, pooled human plasma, and buffer reagents were purchased from Sigma Aldrich (St. Louis, MO). HepG2 cell line was purchased from ATCC (Manassas, VA). HepaRG cell line was purchased from Biopredic International (Saint Grégoire, France). HepaRG additives (ADD711) were purchased from Lonza (Walkersville, MD). Plates containing 21-day Caco-2 monolayers as well as Caco-2 assay buffers were purchased from MB Biosciences (Chestnut Hill, MA). Cells were all authenticated by manufacturers and were passaged for less than 6 months before collecting data for all experiments. All other cell culture materials, cell sorting set-up beads (for blue lasers), and rapid equilibrium dialysis plates (8K molecular weight cutoff) were purchased from Thermo Fisher Scientific (Waltham, MA). Ames’ test kits (Muta-chromoplate™) and rat liver microsomes were purchased from ebpi (Ontario, Canada). MicroFlow^®^ *In Vitro* micronucleus kits were purchased from Litron Laboratories (Rochester, NY). AFB2a-Arg was synthesized and purified according to previous methods published by our lab [29].

### Cell culture conditions

HepG2 cells were cultured in Dulbecco's Modified Eagle Medium (DMEM) high glucose media with 10% fetal bovine serum (FBS) and 1% penicillin-streptomycin. HepaRG cells were cultured using GlutaMAX supplemented Williams’ E Medium with 1% penicillin and a proprietary mixture of supplements provided by Lonza specifically made for HepaRG culturing. To differentiate HepaRG cells, cells were allowed to grow for 2 weeks and then the media was supplemented with 1.7% DMSO. Cells were then cultured in this DMSO-containing media for an additional 2 weeks to complete differentiation.

### High performance liquid chromatography-mass spectrometry (HPLC-MS) conditions

Chemical analysis was performed using an Agilent 1200 series high performance liquid chromatograph (HPLC) with an on-line Agilent 1200 series diode array detector connected to an Agilent 6220 time-of-flight mass spectrometer. The HPLC was equipped with an Agilent Zorbax Eclipse Plus C18 column (3.5 μm, 2.1 × 150 mm) held at 35°C. Mobile phases of water (solvent A) and 50:50 methanol:acetonitrile (solvent B), each containing 1% formic acid, were used at a flow rate of 0.25 mL/min. Gradient programming was as follows: 20% B at 0 min, ramp to 75% B at 4 min, and ramp to 100% B at 9 min. The diode array collected absorbance between 220 and 900 nm throughout each run. Injection volume for all samples was 3 μL unless otherwise stated. Mass spectrometry data was collected in positive ionization mode with a nebulizer pressure of 35 psi and a capillary voltage of 3300 V. Data collection and analysis was performed using Agilent MassHunter software.

### AFB1 transformation using organic acids and arginine

Mixtures of AFB1 and aflatoxin M1 (AFM1) were prepared in acetonitrile (300 and 250 ng/mL respectively). Additionally, four stock solutions of 10 mg/mL L-arginine were prepared in deionized water. Three of these aqueous solutions also contained citric acid (1 M), phosphoric acid (1 M), or a combination of citric and phosphoric acid (1 M each). Each of the four stocks were then divided into four containers and then adjusted to a pH of 2, 3, 4, or 5 using HCl and NaOH. Aliquots of 100 μL of the aflatoxin mixture were dried using a nitrogen evaporator and then reconstituted in 100 μL of each of the sixteen stock solutions. After dissolution in the aqueous solutions, each sample was boiled for 20 minutes and then injected into the HPLC-MS and analyzed for loss of AFB1 and formation of AFB2a-Arg using AFM1 as an internal standard. All samples were repeated in triplicates. Statistical analysis was performed using two-way ANOVAs, one-way ANOVAs, and Students’ *t*-tests.

### Detoxification of spiked corn samples

Dry corn kernels were divided into three groups containing 2-3 grams of material. Corn was submerged in an acetonitrile solution containing AFB1. The amount of AFB1 solution was adjusted for each group to give 100 ng of AFB1 per gram of corn. The acetonitrile was evaporated under nitrogen gas to give AFB1-spiked corn samples. After spiking, the first group was submerged in the treatment solution (1 M citric acid, 1 M phosphoric acid, 10 mg/mL arginine, pH 3), the second group was submerged in deionized water, and the third group did not receive any solution. The group that received the treatment solution was boiled for 20 minutes to mimic the transformation procedure provided earlier. The supernatant was collected for groups containing treatment solution or deionized water, spiked with 100 ng of aflatoxin B2 (AFB2) as an internal standard, and extracted using Resprep C18 solid phase extraction (SPE) cartridges. SPE cartridges were conditioned with 3 mL methanol and equilibrated with 3 mL deionized water. Samples were loaded onto cartridges at a rate of 1–2 drops/sec. After drying under vacuum for 10 min, cartridges were eluted with 1 mL methanol and then analyzed by HPLC-MS to determine the aflatoxin profile in the supernatants. Solid corn kernels from all three groups were extracted by adding 1 mL methanol containing 100 ng AFB2 and were vortexed for 10 sec. Methanol extracts were analyzed by HPLC-MS to determine aflatoxin species that remained on the corn kernels. A recovery factor was determined based on the untreated spiked samples and was used to correct for sample loss. Blank corn samples were also included for each group to subtract any background signal. All samples were repeated in triplicates. Statistical analysis was performed using one-way ANOVAs, and Students’ *t*-tests.

### Stability of AFB1 and AFB2a-Arg in biological fluids

Simulated gastric and intestinal fluids were prepared according to U.S. Pharmacopeia formulations and pooled human plasma was purchased from Sigma Aldrich. AFB1 and AFB2a-Arg was spiked into all three fluids to give a final concentration of 10 μM. Samples were analyzed by HPLC-MS at time zero (t_0_) and again at a final timepoint (t_f_) for each fluid (gastric t_f_ = 1.5 hours, intestinal t_f_ = 6 hours, plasma t_f_ = 18 hours). Because of high protein content in plasma samples, 25 μL of plasma was mixed with 25 μL of cold acetonitrile containing 5 μg/mL AFB2 and then centrifuged at 4°C at 13,000 g for 5 min to obtain a cleaner supernatant to inject into the HPLC-MS for both t_0_ and t_f_ samples. Percent loss of AFB1 or AFB2a-Arg was calculated for each biological fluid. All samples were performed in triplicates. Statistical analysis was performed using one-way ANOVAs, and Students’ *t*-tests.

### Microsomal stability

Rat-liver extracts and S9 activation reagents were purchased from ebpi and an S9 activation mixture containing MgCl_2_, KCl, glucose-6-phosphate, NADP, phosphate buffer, sterile distilled water, and rat-liver extract was prepared according to the manufacturer's protocol. AFB1 or AFB2a-Arg were dissolved in the S9 mixture or PBS to give a final concentration of 40 nM. Samples were incubated for 4 hours (t_f_) at 37°C. Samples were analyzed by HPLC-MS at t_0_ and t_f_ to determine percent loss of each parent compound. Metabolite formation was identified by UV signals at each compound's respective maximum absorption wavelengths (AFB1: 365 nm, AFB2a-Arg: 347 nm) and confirmed by accurate mass data by mass spectrometry. All samples were performed in triplicates. Statistical analysis was performed using one-way ANOVAs, and Students’ *t*-tests.

### Determination of plasma protein binding by rapid equilibrium dialysis (RED)

AFB1, AFB2a-Arg, propranolol (high binding control), and atenolol (low binding control) were each spiked into pooled human plasma to give individual solutions with a final concentration of 10 mM (<1% DMSO). Spiked plasma samples (200 μL) were dispensed into the sample chamber and PBS (350 μL) was dispensed into the buffer chamber of pre-conditioned, single-use RED plates. Plates were covered with sealing tape and placed on an orbital shaker set to 37°C and shaken at 300 rpm for 4 hours. Seals were removed and 50 μL of each compartment for each compound was removed and placed into centrifuge tubes. To each tube, 50 μL of cold acetonitrile containing AFB2 (5 μg/mL) was added to precipitate proteins and release analytes. Samples were then centrifuged at 13,000 g for 5 min at 4°C. Supernatants were then injected into the HPLC-MS with injection volumes of 2 μL. All samples were performed in triplicates. Statistical analysis was performed using one-way ANOVAs, and Students’ *t*-tests.

### Octanol-water partition coefficient measurement

Individual standards of AFB1, AFB2a, and AFB2a-Arg were prepared in deionized water (pH 7) at a concentration of 300 ng/mL each. Aliquots of 300 μL of each aqueous standard were added to 300 μL of 1-octanol. Solutions were shaken at 1800 rpm for 5 min and then allowed to equilibrate for an additional 30 minutes. Aqueous and organic layers for each sample were separated and analyzed by HPLC-MS. Octanol-water partition coefficients (P) were calculated as follows:

$$P=\frac{\left[\text{Peak area in octanol fraction}\right]}{\left[\text{Peak area in aqueous fraction}\right]}$$

All samples were performed in triplicates. Statistical analysis was performed using one-way ANOVAs, and Students’ *t*-tests.

### Caco-2 permeability assay

Assay plates containing 12 wells of 21-day Caco-2 monolayers were provided by MB Biosciences. Monolayer integrity was verified using transepithelial electrical resistance measurements (TEER) prior to assay. Treatment solutions of AFB1, AFB2a, and AFB2a-Arg were prepared in the provided Hank's buffered salt solution (HBSS) with a pH of 7.4 and a final DMSO concentration of 0.2%. Caco-2 monolayers were first rinsed with HBSS and then the apical chamber was filled with 500 μL of test compound solution while the basal chamber was filled with 1500 μL of blank HBSS (both chambers were kept at pH 7.4). Plates were incubated at 37°C for 2 hours to allow compound equilibration. Compound concentration was measured at *t* = 0 hrs and *t* = 2 hours by HPLC-MS. Permeability (*P*_app_) was calculated as follows:

$$P_{\text{app}}=\frac{\text{dQ}}{\text{dt}}\times \frac{1}{A\times C_{0}}$$

Where dQ/dt is the amount of translocated material over incubation time (nmol/s), A is the area of the insert (cm^2^), and C_0_ is the initial concentration of the test compound applied. Integrity of the monolayers was further verified with an atenolol control group which gave an acceptable *P*_app_ of < 0.5 × 10^6^ cm/s. The test was also repeated in the reverse direction (basal chamber to apical chamber) to determine the efflux ratio (ER). ER was calculated as follows:

$$\text{ER}=\frac{P_{\text{app}(\text{Basal}\to \text{Apical})}}{P_{\text{app}(\text{Apical}\to \text{Basal})}}$$

All samples were performed in triplicates. Statistical analysis was performed using Students’ *t*-tests.

### Ames’ test for mutagenicity

A lyophilized mutant strain of *Salmonella typhimurium* (TA100) was purchased from ebpi along with all Ames’ test reagents. Bacteria were rehydrated in nutrient broth the evening before the assay and allowed to incubate in an incubator held at 37°C for 16–18 hours. Individual solutions of AFB1 and AFB2a-Arg were prepared in sterile water with a DMSO concentration of 0.1%. A reaction mixture containing Davis-Mingoli salts, D-glucose, bromocresol purple, D-biotin, and L-histidine was prepared according to kit protocol and mixed with the test compounds. Additionally, an S9 mixture containing MgCl_2_, KCl, glucose-6-phosphate, NADP, phosphate buffer, sterile distilled water, and rat-liver extract was also prepared according to kit protocol and added to each reaction tube. Lastly, each test solution received 5 μL of bacteria broth culture and was mixed thoroughly. The reaction mixtures containing test compounds and bacteria were dispensed into 96-well plates, sealed, and allowed to incubate at 37°C for 5 days. Both AFB1 and AFB2a-Arg were each evaluated at 10, 30, and 50 ng/well along with a vehicle control. A blank group containing only kit reagents without treatment compounds or bacteria was added to ensure no contamination had occurred at any point throughout the assay. Each treatment group was dispensed into 48 wells. The number of positive wells (either yellow or turbid) was counted for each treatment group as a measure of mutagenicity. All treatments were performed in triplicates. Statistical analysis was performed using a chi-squared test according to previous methods [30] as per manufacturer's recommendation.

### DNA adduct identification

Aliquots of individual solutions of AFB1 and AFB2a-Arg (100 μL, 40 nM each) were dried under nitrogen and reconstituted in 100 μL of a 2.0 mg/mL solution of calf thymus DNA. Either the S9 mixture described earlier (100 μL) or PBS (100 μL) was added to each test compound and all samples were incubated at 37°C for one hour. Afterwards, 600 μL of cold ethanol was added to all tubes to precipitate DNA. The supernatant was discarded and 120 μL of 0.15 M HCl was added to all tubes and then samples were incubated for 37°C for 5 min. All samples were then injected into the HPLC-MS with an injection volume of 12 μL. Aflatoxin-adducts were qualitatively identified by monitoring each treatment compound at their respective maximum UV absorption wavelengths (AFB1: 365 nm, AFB2a-Arg: 347 nm) and confirmed by accurate mass data by mass spectrometry.

### Analysis of micronuclei formation and cytotoxicity by flow cytometry

HepG2 and differentiated HepaRG cells were seeded in 96-well plates at a density of 3.0 × 10^4^ cells/cm^2^, allowed to reattach for 24 hours, and then treated with either AFB1 or AFB2a-Arg for 24 hours in serum free media. Concentrations of test compounds began at 60 μM and were diluted 1:2 with serum free media to 0.9375 μM (DMSO concentration was 0.2%) along with a vehicle treated group for each cell type. Treatment solutions were aspirated and cells were allowed to recover in serum-containing media for 72 hours which is equal to 1.5–2.0 doubling times for both cell lines. The procedure for staining cells was provided in the kit provided by Litron Laboratories which was based off of previous methods [31, 32]. Briefly, plates were placed on ice and media was aspirated. Cells were then stained with Ethidium monoazide (EMA) and placed near a light source for 30 min. Cells were protected from light and then lysed and stained with SYTOX Green. Cell sorting beads were also added to each well in equal amounts. Cells were analyzed using a Becton Dickinson (BD) LSR II analytical flow cytometer equipped with a BD high throughput sampler for 96 well plates. Data was collected using FACSDiva software version 8.0.1. Instrument settings and gating were performed according to kit protocol. Relative cell viability was determined by comparing the ratio of nuclei to beads of treated groups to the vehicle control. Percent micronuclei were measured for cells positive for SYTOX Green and negative for EMA. Micronuclei were only measured in treatment groups with a viability >25%. All treatments were performed in triplicates. Statistical analysis was performed using two-way ANOVAs, one-way ANOVAs, and Students’ *t*-tests.

## Abbreviations

AFB1: aflatoxin B1
AFB2a: aflatoxin B2a
AFB2a-Arg: aflatoxin B2a-arginine
AFBO: aflatoxin-exo-8,9-epoxide
AFM1: aflatoxin M1
HCC: hepatocellular carcinoma
HPLC-MS: high performance liquid chromatography-mass spectrometry
RED: rapid equilibrium dialysis.
